# Does Body Mass Index (BMI) Affect the Reconstruction of Biomechanical Parameters in Patients Undergoing Total Hip Replacement (THR) through the Direct Anterior Approach (DAA)?

**DOI:** 10.3390/jcm13020467

**Published:** 2024-01-15

**Authors:** Manuel Weißenberger, Tizian Heinz, Dominik Rak, Ioannis Stratos, Philip Mark Anderson, Martin Lüdemann, Konstantin Horas, Axel Jakuscheit, Maximilian Rudert

**Affiliations:** Department of Orthopaedic Surgery, University of Wuerzburg, Koenig-Ludwig-Haus, Brettreichstr 11, 97074 Wuerzburg, Germanyi-stratos.klh@uni-wuerzburg.de (I.S.); p-anderson.klh@uni-wuerzburg.de (P.M.A.); m-luedemann.klh@uni-wuerzburg.de (M.L.); k-horas.klh@uni-wuerzburg.de (K.H.); a-jakuscheit.klh@uni-wuerzburg.de (A.J.);

**Keywords:** direct anterior approach (DAA), total hip arthroplasty (THA), total hip replacement (THR), femoral offset, abductor lever arm, obesity

## Abstract

Objective: Direct anterior approach total hip replacement (DAA-THR) is gaining increased interest due to its tissue-sparing nature and rapid recovery. Obesity has been shown to be a significant parameter influencing cup positioning in DAA-THR. It was the intention of this retrospective study to examine how obesity would influence the restoration of native hip biomechanical parameters during DAA-THR. Materials and Methods: A total of 74 patients from a high-volume university orthopedic center after unilateral DAA-THA were included. Patients were retrospectively allocated to a study group (BMI > 30 kg/m^2^) and a control group (BMI < 30 kg/m^2^). Furthermore, propensity-score matching for baseline parameters was performed, leaving 30 patients in each group. Biomechanical parameters of the hip (i.e., femoral offset (FO), abductor lever arm (ABL), acetabular offset (AO), center of rotation (COR), stem alignment (SA), body weight lever arm (BWL), cup inclination (CI), and leg length discrepancy (LLD) were evaluated on standardized plain radiographs, and parameters were compared to the native contralateral hip. Results: Mean BMI in the study group was 35.07 ± 5.13 kg/m^2^ and 25.43 ± 2.64 kg/m^2^ in the control group. There was a significant decrease of the ABL only in the study cohort (*p* = 0.01). CI and SA did not differ between both cohorts. FO was slightly increased compared to the native hip in both groups. There was a marginally higher but non-significant proportion of improper FO restoration in the study group (19 vs. 16 patients, *p* = 0.60). Conclusions: Obesity, as quantified by BMI, only has a limited impact on the adequate reconstruction of native biomechanical parameters of the hip during DAA-THR. ABL was the only parameter to be significantly decreased in the overweight patients after DAA-THR. Therefore, special care should be taken on proper acetabular reaming and consequent seating of the cup in the obese patient to avoid excessive lateral positioning.

## 1. Introduction

Primary total hip replacement (THR) has become a very successful and safe procedure for alleviating pain and restoring patient mobility in cases of end-stage osteoarthritis (OA) of the hip. Because of its high success, THR is commonly referred to as the “operation of the century” [1]. With a projected increase in primary THR of 176% by the year 2040, it is of utmost importance to keep on refining both implants and implantation techniques in an attempt to diminish the burden of associated revision surgeries [2]. In Germany, the projected volume of primary THR is expected to increase by 27% by the year 2040 [3]. While 10-year survival rates, reaching up to 96%, have revealed satisfying results after primary THR, patient satisfaction after THR still needs to be improved. Okafor et al. have shown that a total of 7% of patients remain dissatisfied after THR because of ongoing pain or mechanical problems [4]. Inadequate reconstruction of hip biomechanical parameters (HBP) is thought to be a major contributor to patient dissatisfaction, as incorrect offset reconstruction and high leg length discrepancy (LLD) have been linked to inferior patient-reported outcome measures [5,6]. Jain et al. demonstrated a significant correlation between femoral offset reconstruction (FO) and LLD with the Harris Hip Score [7]. Moreover, correct femoral offset reconstruction has been associated with lower polyethylene wear [8,9] and better total range of motion (ROM), as well as increased abduction strength [10].

With the promotion and gaining popularity of minimally invasive incision techniques, like the direct anterior approach (DAA) [11,12], meticulous reconstruction of HBP may be hampered, as the naturally compromised surgical exposure may hinder correct implant positioning. However, as the DAA is currently still on the rise, with the majority of hip surgeons choosing the DAA as their standard approach in everyday total hip arthroplasty [13], the extent to which this minimally invasive technique may influence proper HBP reconstruction remains obscure. So far, there are only a few studies that have addressed the topic of direct anterior approach total hip replacement (DAA-THR) and its impact on adequate HBP reconstruction, providing vague evidence that HBP can be reconstructed sufficiently using minimal invasive approaches to the hip joint [14,15]. Nevertheless, obesity remains a well-known factor rendering THR a technically challenging and demanding task. It is generally accepted that morbidly obese patients (BMI > 30 kg/m^2^) are more likely to undergo revision surgery because of several factors, which include, but are not limited to, infection and implant failure [16,17]. At the same time, there is a high increase in obesity worldwide, with a projected volume of 65 million obese adults in the US by the year 2030 [18]. Projection models estimate that by the year 2029, 55% of patients undergoing primary THR will be obese or morbidly obese [19]. Thus, it may be a reasonable step to apply the proposed benefits of the DAA (faster recovery rate, reduced early post-operative luxation, and reduced post-operative pain) to the obese patient population seeking THR [20,21,22,23]. Classically, the DAA has long been considered a second choice for primary THR in obese patients [24,25,26]. However, in recent years, the DAA has gained growing interest as a feasible approach, especially in the obese patients, due to reduced fat width localized at the anterior thigh [27]. Thus, several authors have promoted the DAA as their standard approach, regardless of BMI [12,28,29]. Meanwhile, the DAA has also gained sincere interest for one- and two-stage revision arthroplasty [30,31].

So far, the influence of obesity on HBP reconstruction through the DAA has not yet been investigated. As obesity has been linked to impaired restoration of HBP after THR using non-minimally invasive approaches [32,33], we hypothesize that obesity (BMI > 30 kg/m^2^) leads to significantly compromised HBP reconstruction after DAA-THR when compared to non-obese individuals.

## 2. Materials and Methods

### 2.1. Study Population and Implants

A monocentric and retrospective analysis of medical records starting in January 2021 was performed, and until November 2021, a total of 74 patients were found eligible for inclusion. The study was performed in accordance with the ethical standards of the Declaration of Helsinki and approved by the local Ethics Committee (reference nr.20231220 02). Inclusion criteria were defined as follows: (1) unilateral end-stage primary osteoarthritis of the hip joint with subsequent THR, (2) a native hip joint of the contralateral side with none or only mild radiographic signs of OA (Kellgren-Lawrence grade 2 at most), (3) fully available radiographic documentation, and medical records of the individual subject. Patients with secondary OA or with documentation of pelvic, spinal, or lower limb injuries were excluded from the study cohort. The BMI of the patients was routinely recorded the day before surgery and was accessible through the achieved medical records. Two groups were formed based on BMI: group A with a BMI > 30 kg/m^2^ (study group) and group B with a BMI below 30 kg/m^2^ (control group). Surgery was randomly performed by one of five senior orthopedic surgeons. Regarding the implants, uncemented taper (Zimmer Biomet ML-Taper) stems were used. If bone quality was judged as poor by the surgeon and primary press-fit with a cementless stem could not be accomplished, a cemented straight stem was used instead (Zimmer Biomet, Müller straight stem). All patients were treated with an uncemented cup (Zimmer Biomet, Allofit S Alloclassic). Bearing materials were either ceramic with cross-linked polyethylene (Sulox ceramic head—XPE), metal and XPE (Protasul—XPE), or ceramic—ceramic (Biolox delta—Biolox delta). All surgeons were likewise experienced with these implants as they were the standard implants at the department. The DAA was the primary approach to the hip joint in this orthopedic center, and patients were placed supine on a standard surgical table. Incision, preparation, and femoral osteotomy were made according to the techniques described elsewhere [34,35,36,37,38]. Leg length was checked manually intraoperatively by the surgeon by comparing the height of the left and right medial malleoli and the left and right anterior superior iliac spines, both with the trial implants and the final implant in situ. All surgeons aimed to restore native leg length within biomechanical, bony, and soft tissue-related limits. In every case, intraoperative fluoroscopy was used for evaluating and verifying implant position, leg length, and femoral offset.

### 2.2. Radiological Assessment

**Figure 1:**
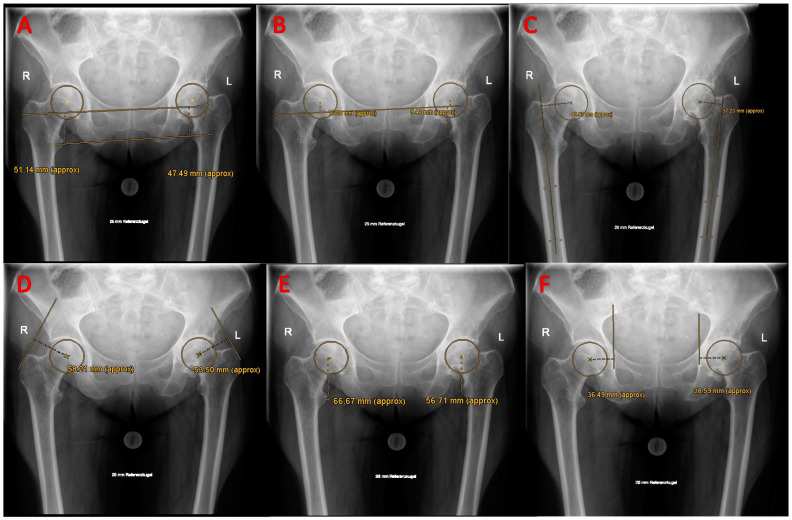
Measurement of biomechanical parameters of the hip: (**A**) leg length difference (LLD); (**B**) vertical hip center of rotation; (**C**) femoral Offset; (**D**) abductor lever arm; (**E**) vertical femoral offset; (**F**) acetabular offset.

Plain radiographs of the pelvis in the anteroposterior view with both legs internally rotated about 15° and another lateral view of the osteoarthritic hip joint were mandatory for every patient prior to surgery. Radiographs were visually evaluated for adequate rotation and tilt of the pelvis prior to templating (i.e., symmetrical obturator foramina, os coccygis, and pubic symphysis on a straight line between one to three cm). A 25 mm radiopaque metal sphere was used for calibration and to assess the degree of magnification for the templating software (MediCAD version 6.0, Hectec Gmbh, Altdorf, Germany). Regardless of the preoperative templates, the final choice of implant size was always made by the surgeon depending on the intraoperative findings. Two to three days following the surgery, radiographs of the pelvis and the hip were repeated in the identical manner. For the evaluation of radiological HBP, the following parameters were evaluated on both the arthroplasty side (termed postoperative) and native contralateral side [14,39] (termed preoperative) (Figure 1): (1) Femoral offset (FO), (2) Acetabular offset, (3) Global offset (femoral offset + acetabular offset), (4) Vertical femoral offset, (5) Abductor lever arm, (6) leg length difference (LLD), (7) Vertical hip center of rotation, and (8) Body weight lever arm (BWLA). Furthermore, the cup inclination and stem alignment angle were measured on every postoperative radiograph. Femoral offset reconstruction was considered adequate if within ±5 mm of the contralateral native hip [40]. Proper cup inclination was defined according to the range proposed by Lewinnek et al. [41], and a leg length discrepancy smaller than 10 mm was considered acceptable [42].

### 2.3. Statistical Analysis

Statistic calculations were performed using SPSS statistical software (SPSS, Chicago, IL, USA, Version 27). For categorial variables, absolute and relative frequencies were calculated. Ordinal variables were expressed as mean values and standard deviations. Data were checked for normal distribution using the Kolmogorov–Smirnov test. Differences between the study and control cohort were assessed using the independent *t*-test. Frequency differences of categorial variables were compared using the chi-square test. Correlative associations of different variables with the outcome parameter were assessed using multiple linear regression analysis, as well as bivariate Pearson and Spearman correlation tests. A *p*-value of 0.05 was set as the level of significance. A priori sample size calculation was performed based on a postulated small-to-medium effect size of obesity on the restoration of biomechanical hip parameters, aiming for a statistical power of 0.90, which revealed a total sample size of 58 patients [43].

## 3. Results

After applying the propensity score matching method to accommodate differences in baseline characteristics, 30 patients in the obese group (group A, termed study group) were successfully assigned to 30 patients in the non-obese group (group B, termed control group). 14 patients were not considered for further analysis because of a lack of a matching partner. 25 patients (41.70%) were female. The mean age turned out to be 61.92 years, and the mean BMI of the total cohort was 30.33 kg/m^2^. Baseline characteristics of both study cohort are depicted in Table 1.

### 3.1. Reconstruction of Hip Biomechanical Parameters

The mean native FO in the study group was 49.59 mm (±8.17 mm) before surgery and slightly increased to 50.86 mm (±6.55 mm) with THR. Similar findings were seen for the control group with a mean increase of about 2.86 mm (±8.61 mm) regarding FO. No significant differences were observed for the mean native, mean postoperative, and average FO change between the study and control groups (Table 2). In the control group, 16 patients (55.17%) were outside the range of adequate FO reconstruction, whereas in the study group, 19 patients (63.33%) had not adequately reconstructed the postoperative FO. Chi-square testing revealed no statistically significant difference regarding the proportion of FO outliners (X^2^ = 0.41, df = 1, *p* = 0.60). Acetabular offset and global offset are depicted in Table 2.

The abductor lever arm (ABL) decreased from 60.81 mm (±5.27 mm) to 58.18 (±9.37 mm) in the control group and from 62.59 mm (±7.25 mm) to 58.27 mm (±7.99 mm) in the study group. The decrease in ABL turned out to be of statistically significant relevance for the study group (*p* = 0.01) (Table 3).

The vertical center of rotation (vCOR) increased both the control and study groups by an average of 3.67 mm ± 5.54 mm (control group) and 3.78 mm ± 4.48 mm (study group). Native and reconstructed vCOR did not reveal any significant differences in both groups.

With THR, the body weight lever arm (BLWA) decreased in both the control and study groups compared to the native hip. However, the difference between native and reconstructed hip was not statistically significant. Reconstructed BLWA was not statistically significant different from native BWLA for both the control and study groups (Table 2).

With respect to LLD, patients in the control group experienced a mean lengthening of 4.43 mm ± 10.53 mm after THR, compared to 7.65 mm ± 8.67 mm in the study group. However, this difference was not statistically significant (*p* = 0.24). A total of 12 hips were shortened with THR (range: 0.16–24.29 mm), and 48 cases experienced a lengthening of the operated leg (range: 0.48–28.85 mm). There were 12 cases in the control group with a postoperative LLD of at least 10 mm compared to 9 cases in the study group, which was not of statistically significant difference (X^2^ = 0.543, df = 1, *p* = 0.57) (Figure 1).

### 3.2. Component Positioning

Mean cup inclination angles were not statistically significantly different in the control and study groups (control: 40.64° ± 5.01°, study: 40.96° ± 3.95°, *p* = 0.78). All cups were positioned within the safe range defined by Lewinnek et al. [41] in both the study and control groups.

Mean stem alignment was 0.83° ± 2.35° of varus in the control compared to 0.85° ± 1.57° of varus in the study group. This difference was not of statistically significant relevance (*p* = 0.98). The centrum collum diaphyseal angle (CCD-Angle) was the only parameter predictive of postoperative stem alignment (spearman r = 0.41, *p* < 0.01).

## 4. Discussion

Obesity in patients needing THR is generally accepted as a challenging and troublesome issue among orthopedic surgeons. This topic becomes even more on the spot when minimally invasive approaches like the DAA are used for THR, allowing for expedited recovery and aesthetically pleasing scars. However, the extent to which the benefits of small-incision and tissue-sparing THR can be transferable to the obese, without compromising postoperative results by limited working space during surgery, is widely unknown. In recent years, a tremendous change has occurred, with several authors promoting the usage of the DAA, especially in obese patient clientele. Classically, the DAA has long been considered unsuitable for primary THA in the obese due to limited exposure and a small working space [24,25,26]. However, data from high-volume centers have shown similar or lower complication rates for the DAA compared to more extensive surgical approaches in obese patient seeking primary THR [28,29]. This finding has raised questions about the limited usage of the DAA solely for normal-weighted patients. Nevertheless, there remains a debate as to whether the DAA may significantly compromise adequate implant positioning, especially in the obese. With the anticipated increase in the number of obese patients seeking primary THR in the coming years, refinement of surgical techniques and approaches seems imperative [19]. Recently, obesity, as measured by BMI, has been linked to a higher risk for cup malpositioning when performed through the DAA [43]. Trevisan et al. were able to demonstrate acceptable reconstruction of HBP a consecutive series of 95 patients undergoing primary THR through the DAA [15]. However, these authors did not specifically consider HBP reconstruction in obese patients. Thus, to the best of the authors’ knowledge, the issue of meticulous native hip anatomy restoration in obese patients by DAA-THR has not yet been studied. Therefore, it was the primary aim of this study to assess how obesity influences the capability of native hip anatomy restoration in patients undergoing DAA-THR.

The reconstruction of both FO and ABL in an attempt to restore native hip anatomy has been shown to be a key factor for successful THR by reducing dislocation rates and early postoperative wear [34,44]. Abduction strength of the abductor muscles is strongly correlated with femoral offset and the abductor lever arm [10]. Therefore, inadequate femoral offset restoration has been shown to negatively affect postoperative functional outcomes and range of motion. Sariali et al. demonstrated that a decrease in native femoral offset by more than 15% alters gait and induces hip instability [45]. Meanwhile, an increase in femoral offset by more than 5 mm has not been linked to better functional outcomes compared to reconstruction to native values [46]. Therefore, reconstruction of the femoral offset within ±5 mm of the native contralateral hip is currently considered proper offset restoration [47,48]. There is vague evidence that femoral offset reconstruction may not be influenced by BMI itself, rather than by the surgical approach [49,50].

The results of this study showed that obesity was associated with a slightly increased number of patients with improper femoral offset reconstruction. However, this effect turned out not to be of statistical significance, and was contrary to the hypothesis that the naturally limited surgical exposure of the DAA would make intraoperative orientation and thus, implant positioning, significantly more difficult in obese patients. There are several factors that may serve as a potential explanation for this non-significant finding. Firstly, there is a very high level of expertise in the minimally invasive DAA at the institution where this study was conducted. The DAA was adopted as the main surgical approach for primary THR at this institution in 2012, and later, indications for the DAA were expanded to include revision surgeries, including one- and two-stage exchanges. The high level of expertise of the senior orthopedic surgeons involved may therefore be a reason for almost identical femoral offset reconstruction in both obese and non-obese patients. Especially for the DAA, a steep learning curve with plateauing revision rates after 100 cases has been well-documented [51,52]. It is therefore reasonable to assume that there is also a learning curve for the DAA in obese patients.

Apart from FO, an interesting finding for the abductor lever arm was noticed. While a non-significant change in femoral offset was seen in both the study and control groups, there was a statistically significant decrease in the abductor lever arm in only the study group. This finding may seem inconsequent at first glance, as a high correlation of femoral offset and the abductor lever arm has been described [46]. However, this is only the case if the center of rotation is not changed. Kurtz et al. have effectively demonstrated the influence of the change of COR on femoral offset and, consequently, on the abductor lever arm [53]. With a superior movement of the COR, the perpendicular distance from the COR to the vector of the gluteus medius muscle decreases, thus indicating a negative correlation between the COR and the abductor lever arm [39]. The abductor lever arm is even more decreased by a superior and lateral movement of the COR, while a superior and medial movement can partially compensate for the loss of abductor moment arm caused by the superior shift.

In this study, the superior movement of the COR was nearly identical for both the study and control groups (study: 3.78 mm ± 4.48 mm vs. control: 3.67 mm ± 5.54 mm). Nevertheless, an additional medial shift (−0.27 ± 5.23) of the COR was seen in the control group, whereas for the study group, an additional lateralization (1.28 ± 5.45) of the COR was observed, which may serve as an explanation for the reduced ABL only in the study group. This finding is indicative of underreaming of the acetabular bed in obese patients, possibly due to impeding soft tissue barriers. Bonnin et al. demonstrated that with conventional THR, a medialization of the cup is routinely performed. The resulting decreased acetabular offset is normally compensated by a higher femoral offset, leading to a restored global offset within 5 mm of the preoperative limits [54].

There are some limitations related to this study. Firstly, this is a retrospective study of prospectively obtained patient data. Prospective randomized research would have added tremendous value to this analysis. However, efforts were made to reduce potential bias by propensity score matching of baseline characteristics for both the study and control groups. We are aware that the number of included patients seems relatively small. However, a priori sample size calculation was performed, and based on a small-to-medium effect size derived from similar studies, a total number of 60 patients seemed sufficient [43]. Correlating the radiographic findings with clinical data would have added additional strength to this study.

So far, very limited studies have investigated the influence of BMI on cup positioning during DAA-THA [43,55]. Al-Amiry et al. were the first to examine the association of BMI with leg-length discrepancy and femoral offset restoration [32]. However, only patients with the anterolateral approach were included in their study, and further biomechanical parameters like ABL and COR were been considered [32]. Further studies have demonstrated an association between the risk of acetabular cup malpositioning and obesity for the minimally invasive approaches [33,56]. Therefore, this is the very first study to explore the association between obesity and hip biomechanical reconstruction parameters using the DAA for THR, building the foundation for further research on this topic.

## 5. Conclusions

- Obesity has a limited effect on the adequate reconstruction of native hip biomechanical parameters during DAA-THR.
- The ABL is the only parameter that significantly decreases in overweight patients after DAA-THR.
- Therefore, special care should be given to proper acetabular reaming and consequent seating of the cup in obese patient to avoid excessive lateral positioning.

## Figures and Tables

**Table 1:** Descriptive statistics of both study groups.

	Study Group (BMI > 30 kg/m^2^)	Control Group (BMI < 30 kg/m^2^)
Sex (female/male)	12/18	13/17
Age (mean, ± SD)	61.37 ± 8.81	62.47 ± 11.15
BMI (mean, ± SD)	35.07 ± 5.13	25.43 ± 2.64
Side of surgery (n, left/right)	20/10	14/16
Cementless stems (n, percent)	28, 93.30%	26, 86.70%
Cementless cups (n, percent)	30, 100%	30, 100%
Median stem size	11.75	11.00
Median cup size (mean, ± SD)	54.00	53.00
Stem with extended offset (n, percent)	8, 26.70%	4, 13.30%

**Table 2:** Characteristics of offset reconstruction for the control and study group at the pre- and postoperative visit.

	Study Group (BMI > 30 kg/m^2^)	Control Group (BMI < 30 kg/m^2^)	*p*-Value (Control vs. Study)
FO (native)	49.59 ± 8.17	48.61 ± 9.51	0.67
FO (reconstructed)	50.86 ± 6.55	52.03 ± 7.65	0.53
Delta FO (reconstructed–native)	1.28 ± 8.44	2.86 ± 8.61	0.48
Number of inadequate FO reconstruction (> 5 mm to native hip)	16 (55.17%)	19 (63.33%)	0.60
AO (native)	40.05 ± 6.10	42.20 ± 6.02	0.18
AO (reconstructed)	41.33 ± 5.86	41.93 ± 7.96	0.74
Delta AO (reconstructed–native)	1.28 ± 5.45	−0.27 ± 5.23	0.27
GO (native)	89.64 ± 10.07	91.61 ± 11.80	0.68
GO (reconstructed)	92.21 ± 8.55	94.17 ± 11.69	0.46
Delta GO (reconstructed–native)	2.56 ± 7.85	2.56 ± 7.52	0.99
BWLA (native)	107.76 ± 5.97	109.78 ± 8.03	0.29
BWLA (reconstructed)	106.84 ± 6.47	107.93 ± 5.15	0.51

**Table 3:** Details of the ABL for the control and study groups.

	Study Group (BMI > 30 kg/m^2^)	Control Group (BMI < 30 kg/m^2^)	*p*-Value (Control vs. Study)
ABL (native)	62.59 ± 7.23	60.81 ± 5.27	0.31
ABL (reconstructed)	58.27 ± 7.99	58.18 ± 9.37	0.97
*p*-value (native vs. reconstructed)	0.01 *	0.12	

* statistically significant at *p* < 0.05.

## Data Availability

Data can be obtained from the authors upon reasonable request.
